# Impact of Multi-Targeted Antiretroviral Treatment on Gut T Cell Depletion and HIV Reservoir Seeding during Acute HIV Infection

**DOI:** 10.1371/journal.pone.0033948

**Published:** 2012-03-30

**Authors:** Jintanat Ananworanich, Alexandra Schuetz, Claire Vandergeeten, Irini Sereti, Mark de Souza, Rungsun Rerknimitr, Robin Dewar, Mary Marovich, Frits van Griensven, Rafick Sekaly, Suteeraporn Pinyakorn, Nittaya Phanuphak, Rapee Trichavaroj, Wiriya Rutvisuttinunt, Nitiya Chomchey, Robert Paris, Sheila Peel, Victor Valcour, Frank Maldarelli, Nicolas Chomont, Nelson Michael, Praphan Phanuphak, Jerome H. Kim, on behalf of the RV254/SEARCH 010 Study Group

**Affiliations:** 1 South East Asia Research Collaboration with Hawaii, Pathumwan, Bangkok, Thailand; 2 The Thai Red Cross AIDS Research Centre, Pathumwan, Bangkok, Thailand; 3 HIV-NAT, Pathumwan, Bangkok, Thailand; 4 Department of Medicine, Faculty of Medicine, Chulalongkorn University, Pathumwan, Bangkok, Thailand; 5 Department of Retrovirology, Armed Forces Research Institute of Medical Sciences – United States Component, Bangkok, Thailand; 6 United States Military HIV Research Program, Rockville, Maryland, United States of America; 7 Vaccine and Gene Therapy Institute, Port St. Lucie, Florida, United States of America; 8 Clinical and Molecular Retrovirology Section/Laboratory of Immunoregulation, Bethesda, Maryland, United States of America; 9 Virus Isolation and Serology Laboratory Applied and Developmental Research Directorate Science Applications International Corporation-Frederick, Inc. National Cancer Institute - Frederick Cancer Research and Development Center, Frederick, Maryland, United States of America; 10 Department of Disease Control, Thailand Ministry of Public Health – United States Centers for Disease Control and Prevention Collaboration, Ministry of Public Health, Amphur Muang, Nonthaburi, Thailand; 11 Memory and Aging Center, University of California at San Francisco, San Francisco, California, United States of America; 12 In Vivo Biology Group, National Institutes of Health, Magnuson Clinical Center, Bethesda, Maryland, United States of America; University of New South Wales, Australia

## Abstract

**Background:**

Limited knowledge exists on early HIV events that may inform preventive and therapeutic strategies. This study aims to characterize the earliest immunologic and virologic HIV events following infection and investigates the usage of a novel therapeutic strategy.

**Methods and Findings:**

We prospectively screened 24,430 subjects in Bangkok and identified 40 AHI individuals. Thirty Thais were enrolled (8 Fiebig I, 5 Fiebig II, 15 Fiebig III, 2 Fiebig IV) of whom 15 completed 24 weeks of megaHAART (tenofovir/emtricitabine/efavirenz/raltegravir/maraviroc). Sigmoid biopsies were completed in 24/30 at baseline and 13/15 at week 24.

At baseline, the median age was 29 years and 83% were MSM. Most were symptomatic (87%), and were infected with R5-tropic (77%) CRF01_AE (70%). Median CD4 was 406 cells/mm^3^. HIV RNA was 5.5 log_10_ copies/ml. Median total blood HIV DNA was higher in Fiebig III (550 copy/10^6^ PBMC) vs. Fiebig I (8 copy/10^6^ PBMC) (p = 0.01) while the median %CD4+CCR5+ gut T cells was lower in Fiebig III (19%) vs. Fiebig I (59%) (p = 0.0008).

After 24 weeks of megaHAART, HIV RNA levels of <50 copies were achieved in 14/15 in blood and 13/13 in gut. Total blood HIV DNA at week 0 predicted reservoir size at week 24 (p<0.001). Total HIV DNA declined significantly and was undetectable in 3 of 15 in blood and 3 of 7 in gut. Frequency of CD4+CCR5+ gut T cells increased from 41% at baseline to 64% at week 24 (p>0.050); subjects with less than 40% at baseline had a significant increase in CD4+CCR5+ T cells from baseline to week 24 (14% vs. 71%, p = 0.02).

**Conclusions:**

Gut T cell depletion and HIV reservoir seeding increases with progression of AHI. MegaHAART was associated with immune restoration and reduced reservoir size. Our findings could inform research on strategies to achieve HIV drug-free remission.

## Introduction

Three decades after the discovery of antiretroviral therapy (ART), complete eradication of HIV infection has not been achieved except under unique circumstances [1]. A slightly less difficult target may be the long-term, drug-free remission of HIV or functional cure through modulation of immune responses; the basis for therapeutic vaccination approaches that, to date, have not provided evidence of control [2]. Preservation of immune function by preventing the CD4 depletion in acute HIV infection (AHI), which occurs prominently in the gut, may be a prerequisite to achieving functional cure [2]. Gut CD4 T cell destruction and mucosal breakdown are linked to immune activation in chronic HIV – a key driver of chronic CD4 decline [3], [4]. Data on immunologic and virologic events as early as Fiebig stages I to III in humans infected with HIV are lacking; yet, such knowledge could inform the design of preventive HIV vaccines and therapeutics [5], [6].

Definitions of AHI vary by study but generally include persons with HIV viremia in the absence of IgG antibody to HIV proteins [7]. Because of high HIV viremia and infectiousness of acute transmitted-founder viruses [8], AHI subjects are more likely to transmit HIV [9]. Symptomatic AHI, sometimes referred to as acute retroviral syndrome (ARS), is characterized by a flu-like syndrome that coincides with peak HIV viremia, and occurs around three weeks after infection [5].

The best practice for clinical management of AHI is currently unknown. Highly active ART (HAART) instituted during early infection could alleviate CD4 loss, suppress viremia, and limit the size of the latent reservoir. However, there is no conclusive evidence for improved long term clinical outcome, and treatment is not always benign [4], [10]. Therefore, treatment of AHI is optional in guidelines [11]. Theoretically, usage of a CCR5 inhibitor and/or an integrase inhibitor in addition to standard ART in AHI may reduce HIV spread and limit immune damage [10].

HIV circulating recombinant form (CRF) 01_AE, and to a much lesser extent, subtype B are prevalent in Thailand [12]. We investigated the clinical, immunologic and virologic characteristics of AHI Thai subjects as well as the short-term outcomes of using multi-targeted HAART. We hypothesized that 1) Real-time pooled nucleic acid testing (NAT) and sequential enzyme immunoassay (EIA) of high prevalence HIV-seronegative subjects from HIV voluntary counseling and testing centers (VCT) in Bangkok would yield volunteers with AHI that is predominantly non-subtype B, and 2) The use of 5-drug ART will have a significant impact on immunity and HIV viral burden.

This study informs the usage of a novel therapeutic strategy with a CCR5 inhibitor in addition to an integrase inhibitor and reverse transcriptase inhibitors in the earliest clinical stage of HIV infection.

## Methods

**Figure 1:**
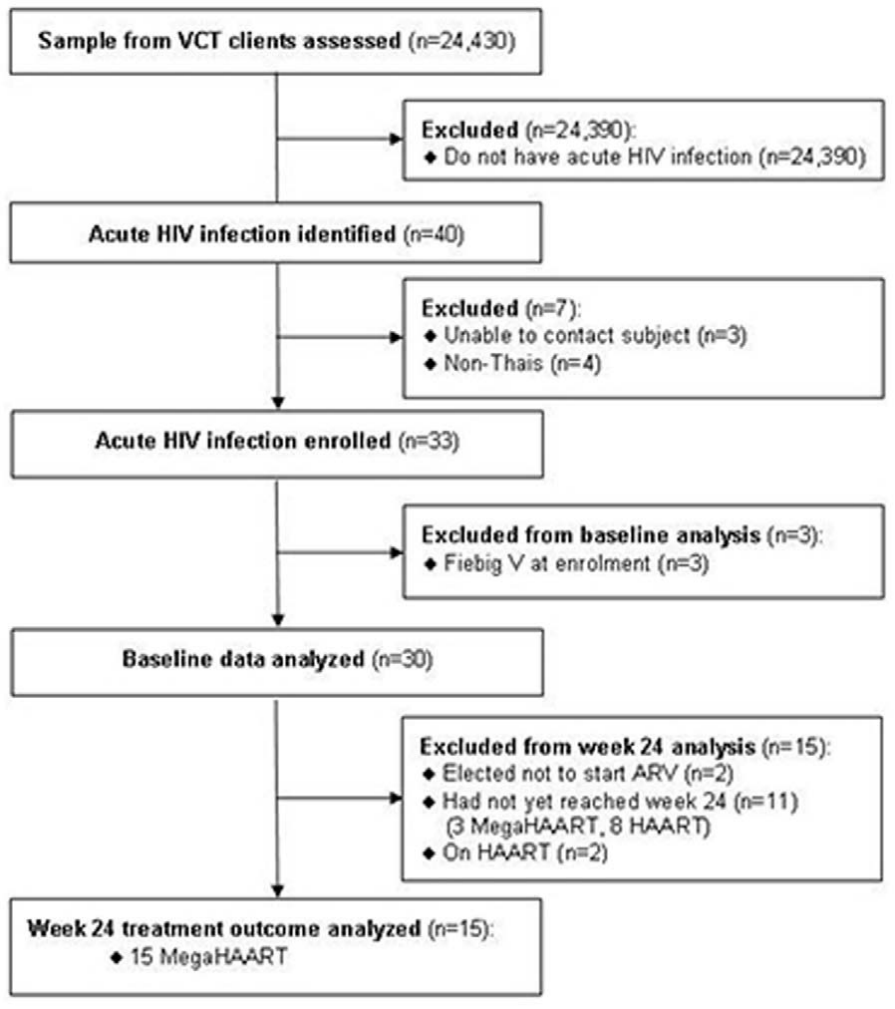
Description of the patient disposition. MegaHAART: Five antiretrovirals (ARVs) including tenofovir, emtricitabine, efavirenz, raltegravir and maraviroc.

The RV254/SEARCH 010 study is an ongoing prospective, open-label study in Bangkok, Thailand (clinicaltrials.gov identification NCT00796146). The study was approved by the institutional review boards (IRBs) of Chulalongkorn University in Thailand and the Walter Reed Army Institute of Research in the United States. All subjects gave informed consent. Samples from subjects who had VCT for HIV at The Thai Red Cross Anonymous Clinic and at the Silom Community Clinic were screened in real-time by pooled NAT and sequential EIA according to published methods [13]. Thai subjects who fit the AHI laboratory criteria for Fiebig stages I to IV [14] were enrolled (Figure 1) and had clinical and laboratory assessments at days 0, 2, 3, 5, 7, 10, weeks 2, 4, 8, 12, 16, 20, 24, and every 12 weeks thereafter up to 192 weeks. A checklist of ARS symptoms was employed by an HIV physician to assess all potential symptoms at the baseline and subsequent visits until all symptoms had resolved.

Laboratory assessments included CD4, HIV RNA, liver transaminases, creatinine, lipids and urinalysis. Plasma and peripheral blood mononuclear cells (PBMCs) were cryopreserved at all visits. Sampling of gut-associated lymphoid tissue (GALT) occurred at weeks 0 and 24 by sigmoidoscopy as an optional procedure (24 subjects at baseline and 13 subjects at week 24), and mucosal mononuclear cells (MMCs) were isolated from GALT.

Initiation of ART was voluntary and done as part of enrollment in an accompanying local protocol (clinicaltrials.gov identification NCT00796263), approved by the Chulalongkorn University IRB, and all subjects gave informed consent. Treatment was started on average 3 days (range 0–5 days) from enrollment. The regimen consisted of 5 antiretrovirals [tenofovir (TDF) 300 mg once daily, emtricitabine (FTC) 200 mg once daily, efavirenz (EFV) 600 mg once daily, raltegravir (RAL) 400 mg twice daily and maraviroc (MVC) 600 mg twice daily] for the first 24 weeks followed by simplification to 3 drugs with TDF, FTC and EFV. EFV was discontinued in subjects with intolerance or resistance, in which case dose adjustment of MVC to 300 mg twice daily was implemented. The first 10 subjects received the 5-drug regimen. Subsequently subjects were randomized 1∶1 (in a block of 30) to 5 drugs (MegaHAART) vs. 3 drugs (HAART with TDF, FTC, EFV) ART.

### Laboratory methods

#### Diagnosis of acute HIV infection (AHI)

All samples were first screened with HIV antigen/antibody combination detection assay EIA (AxSYM; Abbott Laboratories, Wiesbaden, Germany or Roche HIV Combi Assay; Roche Diagnostics, London, UK). Negative samples were screened by pooled NAT using either Roche Amplicor v 1.5 ultrasensitive assay with a lower quantitation limit of 50 copies/ml (Roche Diagnostics, Branchburg, NJ, USA) or Aptima HIV-1 RNA qualitative assay with a lower quantitation limit of 30 copies/ml (Gen-Probe Inc., San Diego, CA, USA). Subjects were confirmed to have AHI if they had positive HIV RNA by another method and had negative non-IgM sensitive (2^nd^ generation) EIA (Genetic Systems rLAV EIA, BioRad Laboratories, Redmond, WA). For samples that were positive for HIV antigen/antibody combination detection assay EIA, the 2^nd^ generation EIA was done immediately. Subjects were confirmed to have AHI if they had negative 2^nd^ generation EIA and also a positive HIV RNA. For Fiebig staging purposes, an IgM-sensitive (3^rd^ generation) EIA (Genscreen HIV 1/2, Bio-Rad, Marnes la Coquette, France), a standard HIV-1 p24 antigen assay (ABL Inc., Kensington, MD) without immune-complex dissociation and Western Blot were done on AHI samples.

AHI subjects were enrolled if they were Thai and fulfilled laboratory criteria for Fiebig stages I to IV as follows: Fiebig I - positive HIV RNA, negative p24 antigen, negative 3^rd^ generation EIA; Fiebig II – positive HIV RNA, positive p24 antigen, negative 3^rd^ generation EIA; Fiebig III - positive HIV RNA, positive p24 antigen, positive 3^rd^ generation EIA, negative western blot; Fiebig IV - positive HIV RNA, positive or negative p24 antigen, positive 3^rd^ generation EIA, indeterminate western blot. All subjects had to have a non-reactive EIA by non-IgM sensitive EIA.

The corresponding mean cumulative durations from onset of HIV infection according to Fiebig et al are 5 (Fiebig I), 10.3 (Fiebig II), 13.5 (Fiebig III) and 19.1 (Fiebig IV) days [14]. For this study, we reported duration from history of HIV exposure within the last 30 days to Fiebig stage diagnosis at screening and at baseline for each subject. For subjects who had multiple dates for possible HIV exposure, the average duration was used.

#### Isolation and Immunophenotyping of PBMCs and sigmoid colon MMCs

PBMCs were cryopreserved in RPMI 1640 medium (Invitrogen, Carlsbad, CA, USA) containing 20% heat-inactivated fetal-calf serum (FCS; Invitrogen, Carlsbad, CA, USA) and 10% dimethylsulfoxide (DMSO; Sigma, St. Louis, MO, USA). For immunophenotyping cryopreserved PBMCs were thawed and washed once in RPMI 1640 media containing 2% heat-inactivated FCS, and 1% penicillin/streptomycin (Invitrogen, Carlsbad, CA, USA), re-suspended in complete RPMI 1640 media and rested over night before staining.

MMCs were isolated from 20–25 pieces of gut-associated lymphoid tissue collected from the sigmoid colon by sigmoidoscopy using Radial Jaw 3 biopsy forceps (Boston Scientific, Natick, MA, USA). The biopsy pieces were placed in complete RPMI 1640 RPMI media containing 10% human AB serum (HAB; Gemini Bio-Product, West Sacramento, CA, USA), 1% HEPES, 1% L-Glutamine, 0.1% Gentamicin (Invitrogen, Carlsbad, CA, USA), 1% Penicillin/Streptomycin and 2.5 µg/ml Amphotericin B (Invitrogen, Carlsbad, CA, USA). Samples were then digested using 0.5 mg/ml Collagenase II (Sigma, St. Louis, MO, USA).Isolated MMCs from one donor were pooled, washed twice and then counted using Trypan Blue exclusion. MMCs were directly used for phenotypical characterization.

**Figure 2:**
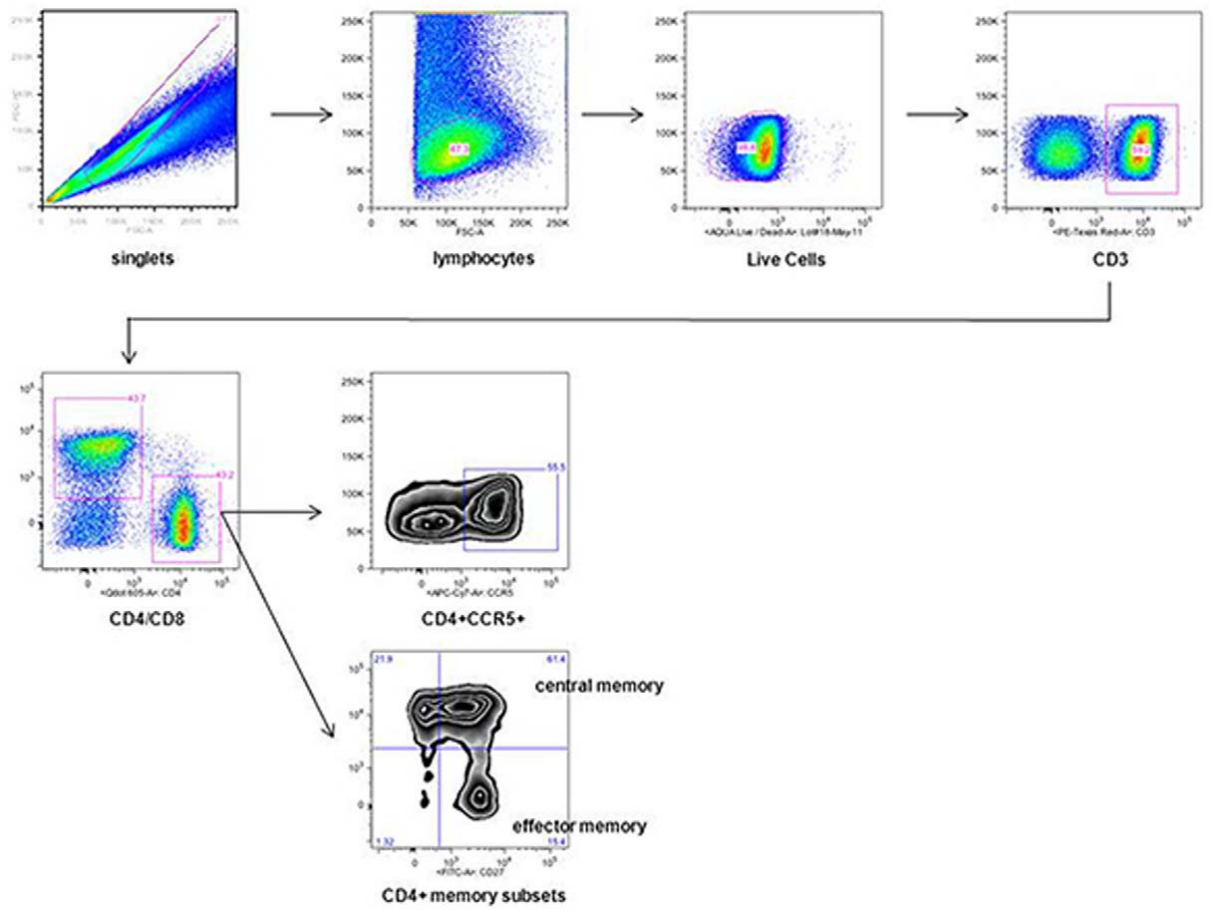
Gating Strategy used to determine the frequency of CD4+CCR5+ T cells. The gating strategy applied for mucosal mononuclear cells (MMC) and peripheral blood mononuclear cells is shown representative on a set of MMC. Initial gating used forward scatter height versus forward scatter area plot to exclude doublets, and forward scatter height versus a sideward scatter height plot to isolate lymphocytes. Dead cells were excluded by Aqua Live/Dead staining and subsequently CD3+ and CD4+ T cells were included. Frequencies of CCR5+ T cells are expressed as frequency of CD4 T cells. Effector memory (EM) T cells were defined as CD27− and CD45RO+ and central memory (CM) T cells as CD27+ and CD45RO+ CD4+ T cells.

Immunophenotyping analysis was performed on cryopreserved PBMCs and freshly isolated MMCs. Cells were first stained with Aqua Live/Dead dye (Invitrogen, Carlsbad, CA, USA) following staining with anti-CD4-QDot605 (Invitrogen, Carlsbad, CA, USA), anti-CD3-PE-TexasRed (Invitrogen, Carlsbad, CA, USA), anti-CD8-V450 (BD Horizon, San Diego, CA, USA), anti-CD27-AlexaFluor700 (BD Pharmingen, San Diego, CA, USA) and anti-CD45RO-PE-Cy7 (BD, San Jose, CA, USA) for 20 min at room temperature. Subsequently cells were washed with PBS and stained with anti-CCR5-APC-Cy7 (BD Pharmingen, San Diego, CA, USA) for 30 min at 37°C. Cells were acquired on a custom built BD LSRII cytometer (BD, San Jose, CA, USA). At least 150,000 total events were acquired in the lymphocyte light scatter gate and the data were analyzed using FlowJo software version 8 or higher (TreeStar, Ashland, OR, USA). Initial gating used forward scatter height versus forward scatter area plot to exclude doublets, and forward scatter height versus a sideward scatter height plot to isolate lymphocytes. Dead cells were excluded by Aqua Live/Dead staining and subsequently CD3+ and CD4+ T cells were included. The gating strategy is shown in Figure 2. Frequencies of CCR5+ T cells are expressed as frequency of CD4+ T cells. Effector memory (EM) T cells were defined as CD27- and CD45RO+ and central memory (CM) T cells as CD27+ and CD45RO+ CD4+ T cells.

#### HIV quantification

At baseline and during follow up, HIV RNA in plasma was performed using Roche Amplicor v 1.5 ultrasensitive assay with a lower quantitation limit of 50 copies/ml (Roche Diagnostics, Branchburg, NJ, USA). For gut tissue, frozen samples were weighed then homogenized in AVL buffer (QIAamp Viral mini kit Cat No. 52,904) using a mini mortar and pestle. Extraction was completed per kit instructions. The Siemens Quantiplex HIV-1 3.0 assay was used to measure HIV-1 RNA copy number. Results were expressed as copies/mg of tissue.

#### Total HIV DNA in gut and blood

Quantifications of total HIV DNA were performed as described previously [15]. Primers and probes have been specifically designed for CRF01_AE and B. Briefly, PBMCs and sigmoid biopsies were digested with proteinase K and lysates were directly used for amplification. A modified nested PCR was used to quantify both HIV DNA and CD3 gene copy numbers. As a standard curve for both quantifications, serial dilutions of ACH2 cells (NIAIDS reagent program) ranging from 3×10^5^ to 3 cells were amplified together with experimental samples. HIV sequences and the CD3 gene were co-amplified for 12 cycles in triplicate wells. PCR products were diluted and HIV and CD3 copy numbers were determined in separate second amplification reactions on the Rotor-gene Q instrument (Qiagen). Data from elite controllers and chronic HIV patients on suppressive therapy were used for comparison. Their samples were run in the same laboratory. Although the primers and probes have been modified from a clade B assay to enable detection of both clade B and recombinant CRF01_AE for this particular study, the limit of detection of both assays is comparable (1 copy per reaction tube). To validate our assay, we amplified the same samples with the 2 assays and saw a strong positive correlation between the assays (r = 0.93, p<0.0001).

#### HIV subtyping/sequencing

HIV-1 infected plasma samples were assigned subtypes using the multi-region hybridization assay, a real-time PCR based assay designed specifically for subtypes B, C and CRF01_AE (MHAbce) utilizing probes to 8 regions throughout the HIV-1 genome [12].

#### Cytokines

Interferon (IFN)-α and interleukin (IL)-17 were measured using a custom Q-Plex IR ELISA Array (Quansys Biosciences, Logan UT). Images were captured using the Odyssey imaging system (Li-Cor, Lincoln NE) and analyzed using Q-View Plus software (Quansys Biosciences). Interferon gamma-induced protein (IP)-10 was measured by standard ELISA (Invitrogen, Carlsbad CA).

#### Biomarkers

Biomarkers were measured in cryopreserved EDTA plasma, D-dimer was measured using an enzyme-linked fluorescent assay on a VIDAS instrument (bioMerieux Inc., Durham, North Carolina, USA). Hyaluronic acid (HA) was measured using HA test kits (Corgenix, Inc, Westminster, Colorado, USA). C-Reactive Protein (CRP) was measured by electrochemiluminescence (Meso Scale Discovery, Gaithersburg, Maryland, USA). Soluble CD14 (sCD14) was measured by ELISA (R&D Systems, Minneapolis, Minnesota, USA). Lipopolysaccharide (LPS) levels were quantified by first diluting fasting plasma samples, collected in EDTA tubes, to 10% with endotoxin-free water and subsequent heat inactivation of plasma proteins for 15 minutes at 80°C. Measurements of the samples were made with a Limulus Amebocyte Assay (Lonza). Samples were measured in duplicate and background was subtracted.

### Statistical analysis

For this publication, data captured between April 2009 and December 2010 of subjects with Fiebig I to IV at enrollment were analyzed. This included baseline information of the first 30 subjects and 24-week treatment outcome data of the first 15 subjects treated with megaHAART (Figure 1). Fifteen subjects were excluded from the 24-week treatment outcome analysis because two had elected not to start ART, 11 had not yet reached week 24 (3 were on megaHAART and 8 were on HAART), and 2 had reached week 24 but were randomized to HAART. The latter two patients were excluded as the goal for the initial phase of the study was to evaluate the impact of megaHAART on immunity and HIV reservoir. At baseline, all variables were analyzed for the 30 subjects. The non-parametric Wilcoxon rank-sum test was used to compare the quantity of HIV DNA in PBMCs as well as other continuous variables between patients by Fiebig stage. Changes in CD4+ T cells, CD4+CCR5+ T cells, HIV RNA, HIV DNA content, plasma cytokines and inflammatory biomarkers from baseline to week 24 in 15 subjects with data for all time points were assessed using the Wilcoxon matched-pairs signed-rank test. The total HIV DNA in PBMCs at baseline was used as a predictor of reservoir size at week 24. Correlation between the two variables was assessed by Spearman's rank correlation coefficient. Statistical tests were 2-sided with p-values<0.05 considered statistically significant.

Statistical analyses were performed using Prism version 5.01 software (Graphpad, software inc.) and STATA/IC version 11.2 for windows (Statacorp LP, TX, USA).

## Results

### Diagnosis of AHI and characteristics of AHI subjects

Between 20 April 2009 to 31 December 2010, 24,430 samples were prospectively screened to identify 40 subjects with AHI (Figure 1). Twenty-six were identified by pooled NAT (negative HIV IgM antibody – Fiebig I/II) and 14 were identified by sequential EIA (positive HIV IgM antibody – Fiebig III/IV) with an overall AHI incidence of 1.7/1000 screened; 95% confidence interval 1–2.7/1000. Seven did not enroll in the study −3 were not able to be contacted and 4 were non-Thais. Thirty-three subjects enrolled in the study but 3 were excluded from the baseline analysis as they had progressed to Fiebig V at enrollment.

Subjects were mostly young MSM with an estimated time of HIV exposure of about two weeks (Table 1). The median CD4 count was 406 cells/mm^3^ and the plasma HIV RNA was 5.5 log_10_copies/ml. The median gut HIV RNA was 596 copies/mg tissue and 6 had levels <50 copies/mg tissue. Most (70%) were infected with HIV-1 CRF01_AE. The seven non-typable samples by MHAbce contained CRF01_AE (n = 3), B (n = 3) and CRF01_AE/B (n = 1) genetic material. Almost 80% had R5 HIV virus by the Trofile assay, and 10% had primary NNRTI resistance. Most (n = 26) were symptomatic with fever, myalgia and fatigue being the most common manifestations, occurring around 11 days after estimated time of exposure (Table 2). Notably absent were respiratory symptoms. The mean duration (SD) from onset of HIV exposure by self-reporting history to Fiebig stages was 12 (9.6) days for I, 16 (5.6) days for II, 18 (7.8) days for III, and 29 (3.6) days for IV.

**Table 1:** Baseline characteristics of acute HIV-infected subjects.

Characteristics	Cohort with baseline data (n = 30)	Subgroup with 24-week mega HAART outcome data (n = 15)
Median (IQR) age, years	29 (25, 32)	29 (25, 30)
Gender Male: Female, n	26∶4	13∶2
Risk behavior, n (%)		
MSM	25 (83)	12 (80)
Heterosexual male	1 (3)	1 (7)
Heterosexual female	4 (13)	2 (13)
Mean (SD) duration since onset of HIV, days	18 (9.1)	15 (8.4)
Fiebig stage, n (%)		
I	8 (27)	3 (20)
II	5 (17)	2 (13)
III	15 (50)	8 (53)
IV	2 (6)	2 (13)
Acute retroviral syndrome, n (%)	26 (87)	12 (80)
Median (IQR) CD4, cells/mm^3^	406 (298, 555)	381 (298, 525)
Median (IQR) plasma HIV RNA, log_10_ copies/ml	5.5 (5.1, 6.4)	5.7 (5.4, 6.4)
Primary drug resistance, n (%)		
NRTI	T215F (n = 1)	None
NNRTI	K103N (n = 2), Y181C (n = 1)	K103N (n = 2), Y181C (n = 1)
PI	None	None
HIV subtype by MHAbce*, n (%)		
CRF01_AE	21 (70)	8 (53)
B	1 (3)	1 (7)
Nontypable	7 (24)	6 (40)
Tropism by Trofile, n (%)		
R5	23 (77)	10 (67)
Dual R5/X4	1 (3)	0
Unable to be amplified	6 (20)	5 (33)

* MHAbce: A multi-region hybridization assay distinguishes subtypes B, C and CRF01_AE [12]. One sample was not done due to low plasma HIV RNA. MSM: Men who have sex with men, NRTI: Nucleoside reverse transcriptase inhibitor, NNRTI: Non-nucleoside reverse transcriptase inhibitor, PI: Protease inhibitor.

**Table 2:** Clinical manifestations of acute retroviral syndrome.

Symptoms	N (%)	Mean onset (range) from HIV exposure, days
Overall	26 (87)	11 (1–20)
Fever	23 (77)	11 (1–20)
Myalgia	18 (60)	10 (1–20)
Fatigue	17 (57)	8 (1–19)
Oral ulcer	16 (53)	13 (6–20)
Skin rash	15 (50)	14 (4–32)
Sore throat	15 (50)	9 (1–22)
Headache	15 (50)	12 (1–20)
Anorexia	11 (37)	12 (1–21)
Diarrhea	11 (37)	10 (1–19)
Arthralgia	8 (27)	9 (1–18)
Nausea and vomiting	5 (17)	15 (10–21)
Adenopathy	4 (13)	17 (11–22)
Weight loss	4 (13)	10 (7–14)

**Figure 3:**
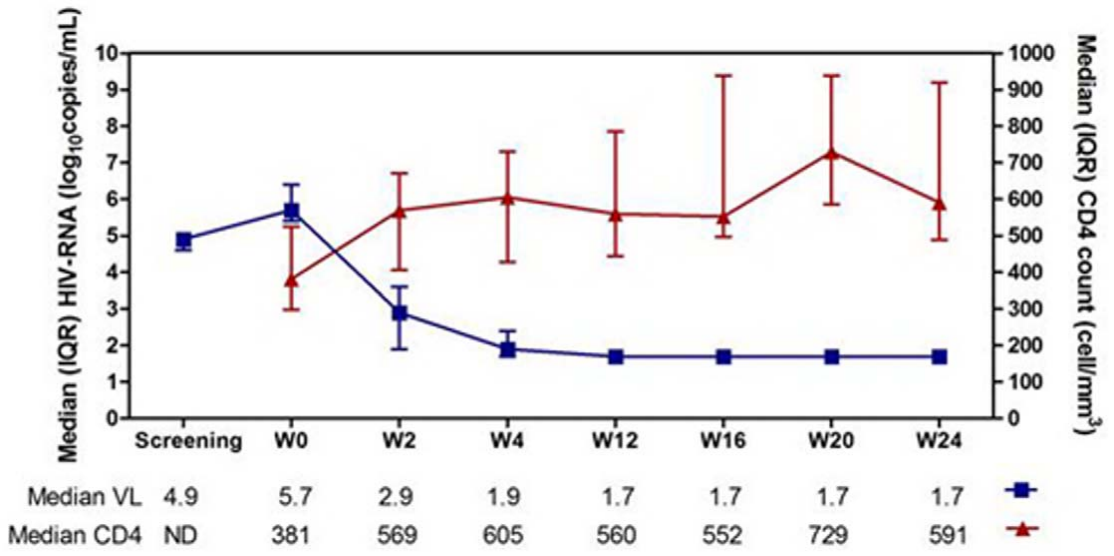
Plasma HIV RNA and CD4 response to megaHAART. The mean time from the screening visit to week 0 is 3 days (standard deviation 1.6 days).

The first 15 subjects on megaHAART had treatment outcome data up to week 24 (Figure 1). They had similar characteristics as the whole cohort (Table 1). Figure 2 shows a rise in HIV RNA between the screening and baseline visits [mean (SD) of 3 (1.6) days] suggesting that our subjects are captured early before reaching peak viremia. After treatment, plasma HIV RNA declined rapidly with 6/15 achieving HIV RNA<50 copies/ml by week 4 and 14/15 having undetectable HIV RNA at week 24 (Figure 3). At week 24, HIV RNA in gut tissue was below 50 copies/mg tissue in all 13 subjects tested. Median peripheral blood CD4 count rose by 188 cells/mm^3^ at week 2 followed by a sustained rise to 591 cells/mm^3^ at week 24. Four discontinued EFV before week 12 due to CNS symptoms (1), rash (1) and primary NNRTI resistance (2).

### Gut T cell depletion during AHI and its restoration following megaHAART

**Figure 4:**
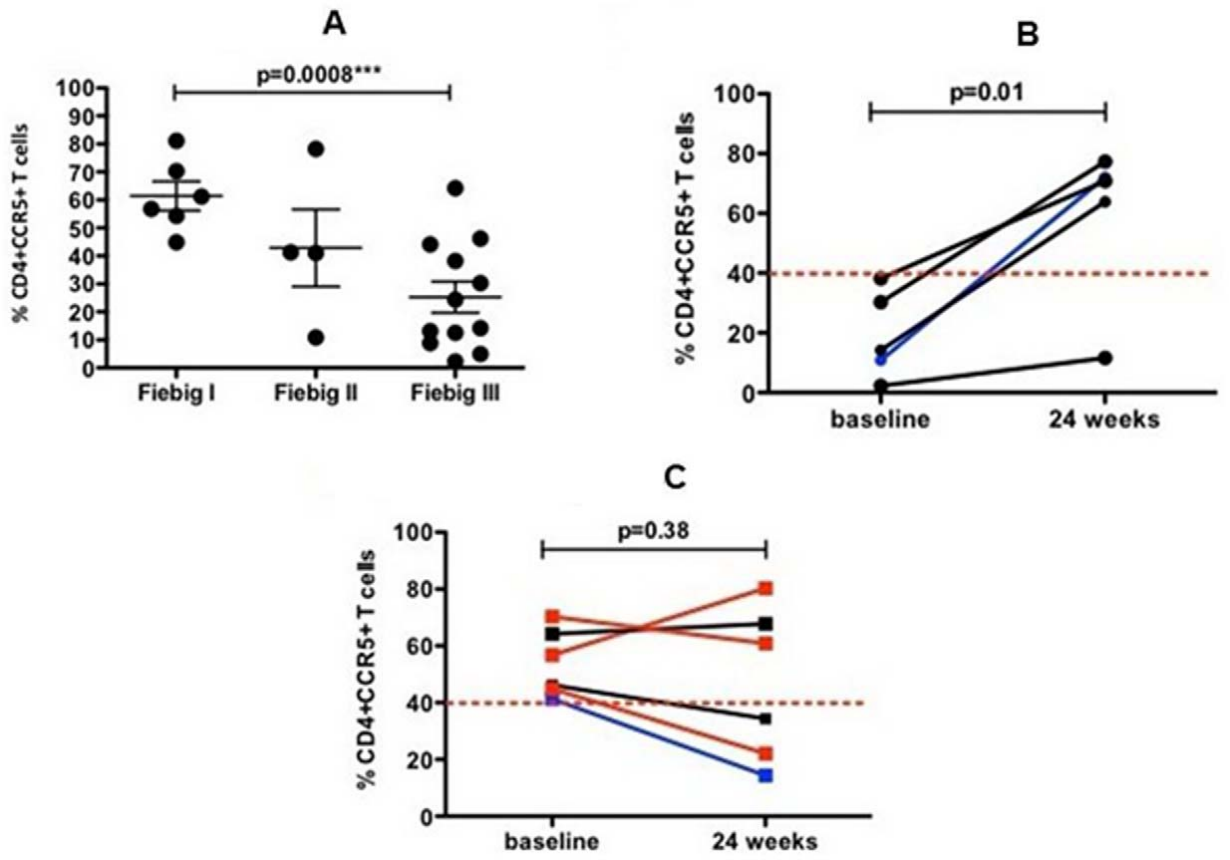
Frequency of CD4+CCR5+ T cells in sigmoid colon in acute-HIV infected subjects. In the sigmoid colon of acute HIV infection patients, the frequency of CD4+CCR5+ T cells declined significantly from Fiebig I (median: 53%) to Fiebig III (median: 19.3%) (p = 0.0008) (**Figure 4A**). After 24 weeks of megaHAART, the frequency of CD4+CCR5+ T cells was restored in patients with a baseline frequency below the median value of 40% (**Figure 4B**) while patients with a frequency above the median value of 40% at baseline showed a variable response to treatment (**Figure 4C**). For Figure 4A, each dot represents an individual subject with horizontal bars showing median values and interquartile ranges. For Figures 4B and 4C, each line represents changes of gut CD4+CCR5+ T cells from baseline to week 24 in an individual subject. Blue line – Fiebig I; black line – Fiebig II; red line – Fiebig III. Data from two Fiebig IV subjects were not included in the analysis.

For the sigmoid biopsy flow analysis, data for 22 subjects at baseline and 11 subjects at week 24 were available for analysis. At baseline, a significant decrease in the median frequency of CD4+CCR5+ T cells with progression of Fiebig stages was observed with 53% at Fiebig I and 19.3% at Fiebig III (p = 0.001) (Figure 4A). The loss of CD4+CCR5+ T cells between Fiebig I and Fiebig III mainly occurred within the effector memory (EM: CD27-CD45RO+) and central memory (CM: CD27+CD45RO+) CD4+ T cell sub-sets. The median frequency of CD4+CCR5+ EM T cells dropped from 56% at Fiebig I to 18% at Fiebig III (p = 0.008) while CM T cells dropped from 80% to 46% from Fiebig I to Fiebig III, respectively (p = 0.001).

This association was not seen for PBMCs, where the frequency of CD4+CCR5+ T cells at Fiebig I was 9.1% (n = 6) compared to 9.6% at Fiebig III (n = 8) (p>0.05). However there was no change in the frequency of CD8+CCR5+ T cells in the gut mucosa (Fiebig I: 91.6%; Fiebig III: frequency 94.6%, p>0.05) that could contribute to the changes observed in the frequency of CD4+CCR5+ T cells. Additionally we observed that in the gut mucosa CD4+CCR5+ T cells depletion increased with higher total HIV DNA, p = 0.05 and r = 0.46.

After 24 weeks of megaHAART, the median frequency of CD4+CCR5+ T cells in the gut mucosa showed an increased trend compared to baseline (baseline: 41%, 24 weeks: 64%, p>0.05). However, in five subjects with a frequency of CD4+CCR5+ T cells below the median baseline value of 40%, a significant increase in the frequency of CD4+CCR5+ T cells from baseline to 24 weeks of treatment of 14% to 71% (p = 0.02), respectively, was observed (Figure 4B). The increase in CD4+CCR5+ T cells was mainly seen in the EM (baseline: 36%, 24 weeks: 82.2%, p = 0.02) and CM subsets (baseline: 18%, 24 weeks: 62.6%, p = 0.003). In contrast, a variable response to treatment was observed in patients with baseline CD4+CCR5+ frequency above the median of 40% (Figure 4C). The same trend (although not statistically significant) could be observed for the actual count of CD4+CCR5+ T cells with those patients having a median baseline value below 40% showing an increase in their CD4+CCR5+ T cell count after treatment (baseline: median 696, 24 weeks: median 7165, p<0.05). Additionally there was no change observed in the frequency and actual count of CD8+CCR5+ T cells in the sigmoid colon (baseline: 93% and median: 16812, 24 weeks: 93% and median: 18320, p>0.05). In the peripheral blood, no significant changes in the frequency of CD4+CCR5+ T cells were observed as a response to 24 weeks of megaHAART (baseline: 9%, 24 weeks: 8.3%, p>0.05).

#### HIV reservoir

**Figure 5:**
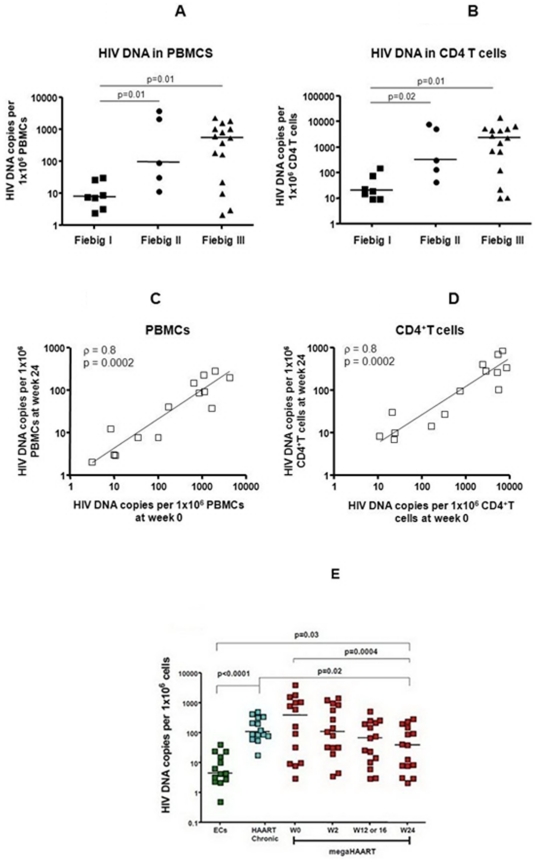
HIV reservoir size by total HIV DNA quantification in peripheral blood mononuclear cells and CD4+ T cells of acute-HIV infected subjects. Footnote: At baseline, the HIV reservoir size, determined by the frequencies of HIV-infected cells expressed as HIV DNA copies per million peripheral blood mononuclear cells (PBMCs) (**Figure 5A**) or calculated per million CD4+ T cells (based on CD4 frequency among PBMCs measured by flow cytometry) (**Figure 5B**), increased with progression of Fiebig stage. The amount of total HIV DNA in PBMCs (**Figure 5C**) and in CD4+ T cells (**Figure 5D**) at baseline predicted the HIV reservoir size after 24 weeks of antiretroviral treatment. The median total HIV DNA in PBMCs of subjects treated during acute HIV infection in this study was lower than that of subjects treated during chronic HIV infection. In addition, some acutely treated subjects achieved HIV DNA in PBMCs as low as that in elite controllers (**Figure 5E**). For all figures, each data point represents an individual subject. For figures 5A to 5D, the horizontal bars represent the median values. For figure 5E, megaHAART (highly active antiretroviral therapy) represents subjects in this study who initiated 5-drug antiretroviral regimen during acute HIV infection (n = 15). Data from two control groups are included here as HAART chronic and ECs (elite controllers). HAART chronic represents subjects who initiated standard antiretroviral therapy during the chronic HIV infection stage and had received treatment for a mean duration of 56 months (n = 14). Elite controllers represent subjects whom HIV RNA are undetectable in the absence of antiretroviral therapy (n = 13).

The size of the HIV reservoir was determined by quantifying the total number of HIV DNA copies per 10^6^ cells in PBMCs and in gut mucosal tissue. Data on HIV DNA in PBMCs were available for 29/30 subjects at baseline and 15/15 at week 24. The total HIV DNA in PBMCs was significantly higher in subjects at Fiebig III (median 550 copies/10^6^ PBMCs, n = 15) and Fiebig II (median 96 copies/10^6^ PBMCs, n = 5) compared to those at Fiebig I (median 8 copies/10^6^ PBMCs, n = 7) AHI, p = 0.01 (Figure 5A). As more than 95% of the reservoir is harbored by CD4+ T cells (Chomont N, unpublished data), we estimated the frequency of infection of CD4+ T cells in the blood based on their percentage among PBMCs (measured by flow cytometry). We saw similar results when infection frequencies in CD4+ T cells were used (Figure 5B) rather than infection frequencies in PBMCs. The median total HIV DNA were significantly higher in Fiebig III (2308.5 copies/10^6^ CD4) and Fiebig II (319.7 copies/10^6^ CD4) compared to Fiebig I (20.5 copies/10^6^ CD4) subjects (p = 0.01 and 0.02 respectively). The total HIV DNA in PBMCs (Figure 5C) and in CD4+ T cells (Figure 5D) at baseline predicted reservoir size at week 24, (*ρ* = 0.8, p = 0.0002). After megaHAART, the median total HIV DNA declined from 1513 copies/10^6^ CD4 at baseline to 106 copies/10^6^ CD4 at week 24 (p = 0.002).

By week 24, acutely treated subjects achieved total DNA levels (median 40 copies/10^6^ PBMCs) lower than those in virologically suppressed, chronically HIV-infected patients on long-term standard HAART (median 109 copies/10^6^ PBMCs, n = 14), and 9 patients had levels as low as those seen in elite controllers who maintain undetectable viremia without ART (median 4.5 copies/10^6^ PBMCs, n = 13) (Figure 5E). Three of 15 acutely treated subjects had undetectable total HIV DNA in PBMCs.

HIV DNA data in gut tissue were available in 19/30 subjects at baseline and 7/15 at week 24. The median total gut HIV DNA was 336 copies/10^6^ cells in Fiebig III (n = 11) and 10 copies/10^6^ cells in Fiebig I (n = 5) (p>0.05). The total HIV DNA in gut among the 7 subjects with week 24 results decreased from 319 copies/10^6^ cells at baseline to 39 copies/10^6^ cells at week 24 (p = 0.047).

#### Plasma inflammatory biomarkers

**Figure 6:**
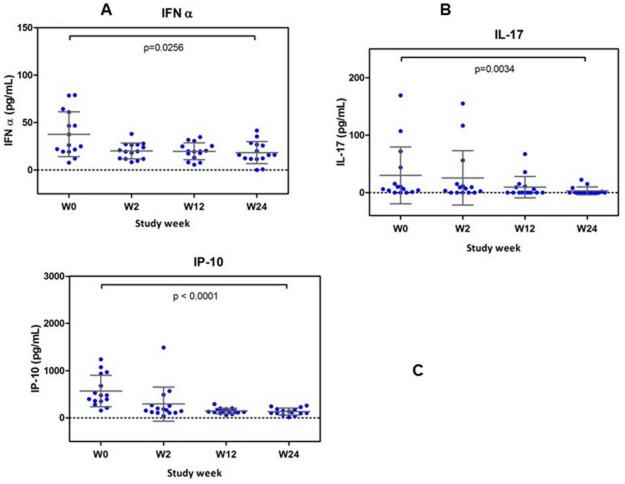
Decline in plasma cytokines following 24 weeks of antiretroviral treatment. Interferon (IFN)-α (**Figure 6A**); interleukin (IL)-17 (**Figure 6B**); interferon gamma-induced protein (IP)-10 (**Figure 6C**). All cytokine levels were significantly reduced following treatment.

**Figure 7:**
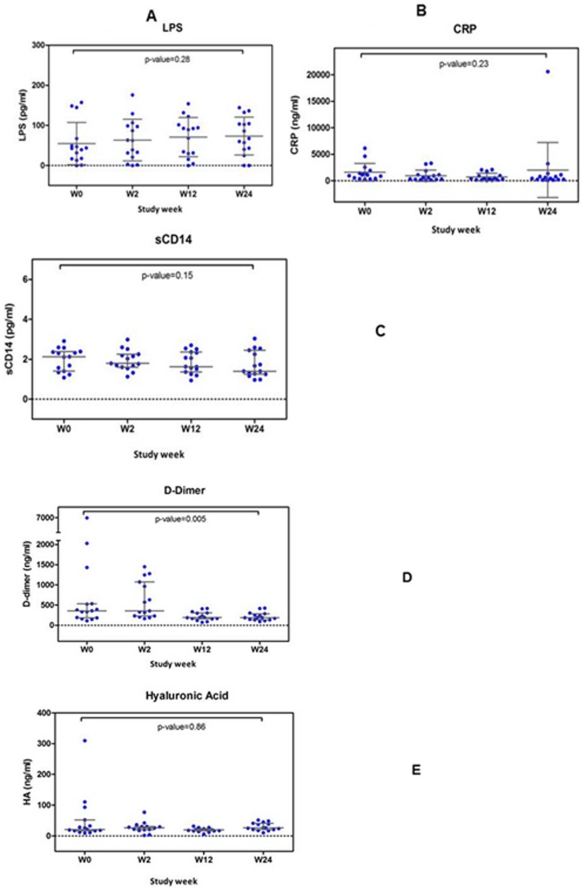
Inflammatory biomarkers following 24 weeks of antiretroviral treatment. Lipopolysaccharide (LPS) (**Figure 7A**); C-reactive protein (CRP) (**Figure 7B**); soluble CD14 (sCD14) (**Figure 7C**); D-dimer (**Figure 7D**) and hyaluronic acid (**Figure 7E**). Only D-dimer levels were significantly reduced after treatment.

Significantly lower levels of plasma IFNα (Figure 6A), IL-17 (Figure 6B) and interferon gamma-induced protein (IP-10) (Figure 6C) were seen following treatment. Of the inflammatory markers measured, only D-dimer showed a significant reduction following 24 weeks of megaHAART whereas plasma LPS, CRP, sCD14 and HA concentrations were not different from baseline (Figures 7A to 7E).

## Discussion

At the incipient stages of HIV infection, there is a massive loss of GALT-associated CD4+ T cells in animal models of simian immunodeficiency virus (SIV) and HIV in humans as a consequence of direct infection [3], [16], [17]. Increased gut permeability and chronic activation of CD4+ T cells by bacterial products further potentiates T cell loss [3], [4], [16]. The clinical course of acute CRF01_AE infection has not been described before. In our study, we identified subjects in the earliest stage of infection (90% were Fiebig I–III) and evaluated the immunologic and virologic changes in both peripheral blood and gut before and after aggressive ART. We demonstrated that gut T cell depletion and HIV DNA reservoir size increased as Fiebig stage progressed, and gut total HIV DNA correlated with the depletion of gut CD4+ T cells. Importantly, the amount of total HIV DNA at entry into the study predicted reservoir size after treatment. These findings favor early intervention during AHI to limit immune destruction and HIV reservoir size, and also highlight that immune destruction begins in the earliest days after infection. The latter finding raises concerns regarding the interpretation of these data in the context of protection when considering treatment in later stages of primary infection [10].

It is thought that the early depletion of the GALT, the largest reservoir of CD4+ T cells in the body, is a blow from which the host may not recover even after prolonged ART in the chronic phase of infection [2], [3], [5], [18]. We employed a strategy that blocked HIV at three steps in the viral life cycle -at entry (CCR5 inhibitor), reverse transcription [nucleoside reverse transcriptase inhibitors (NRTIs) and non-NRTI) and integration (integrase inhibitor)-and found a marked reduction in viral burden in both gut and plasma HIV RNA and DNA. The extent of HIV DNA reduction after 6 months of therapy exceeded that achieved in chronically infected patients following almost 5 years of conventional three-drug ART. Importantly, in persons whose gut CD4+CCR5+ T cells were depleted, megaHAART was associated with reconstitution of gut CD4+CCR5+ T cells to the normal range. This has not been described in published data of macaques or humans treated with standard HAART which may reflect ART timing and/or regimen [17], [18], [19], [20]. Lower CCR5 may reduce the replication of HIV by the fusion inhibitor enfurvitide, and a similar mechanism may be at work for MVC [21]. CD4+ CM T cells are preserved in elite controllers and natural SIV hosts suggesting that maintaining CD4+ CM T cells and limiting HIV integration may be crucial to achieving drug-free control of HIV replication [2], [5], [22]. Our data showed that both CM and EM CD4+CCR5+ T cells are predominantly depleted in the gut during AHI, and institution of ART during Fiebig I to III reconstituted these subsets. Decreased IFNα and IL-17 levels following treatment may be a consequence of the diminished viral reservoir leading to reduced immune activation, but further studies are needed [23]. Damage to the gut mucosal barrier leading to microbial translocation has been proposed as a cause of ongoing immune activation despite successful ART in chronic HIV infection reflected by elevated inflammatory biomarkers such as those investigated in our study [24], [25]. These markers have been linked to HIV progression and complications in chronic HIV infection [26], [27]. In contrast to the cytokine rise seen prior to peak viremia [28], elevated levels of markers of gut microbial translocation have not been consistently reported in the acute HIV infection period [5]. Our patients were enrolled in early acute HIV infection when significant gut microbial translocation may have not yet occurred. Supporting evidence were the lower levels of inflammatory biomarkers in our patients compared to those reported in chronically HIV-infected Thais [29], and the lack of significant reductions, except for D-dimer, following treatment despite a profound decline in plasma viremia [30].

Results have been mixed with respect to the benefit of initiating treatment during AHI [10], possibly owing to varying definitions of AHI and timing of ART initiation. In our study, patients initiated treatment in the earliest stage of infection, most at Fiebig stages I–III. Further, we used an aggressive approach with five antiretrovirals, affecting both HIV entry and integration. The integrase inhibitor accelerates HIV viremic decay [31], and the CCR5 inhibitor could be beneficial in AHI since transmitted/founder HIV virurses are almost exclusively CCR5-tropic [32]. A randomized comparison of megaHAART versus standard HAART to discern the benefit of using these additional drugs is now being implemented in our study, and is the subject of ongoing investigation by others [33].

AHI subjects are 20–30 times more likely than their chronically-infected counterparts to transmit infection [8], [32], and early treatment could avert new infections [10]. Our study provided evidence that identifying AHI subjects by NAT and sequential EIA, and enrolling them in a study is feasible but technically and logistically challenging and costly[7], [34]. Strengthening the awareness of symptomatic AHI is a less challenging way to identify acute and recent HIV infection making early treatment far more possible.

Our study has limitations such as the relatively small number of subjects with AHI despite screening a large number of VCT clients and the lack of comparator groups, namely, a group in which treatment was not instituted and a group that received standard HAART. This limits our interpretation of the effect of megaHAART on HIV reservoir size and immune restoration. Additionally the gut biopsy was done at the sigmoid site only. Yukl et al reported differences in cells harboring HIV DNA across gut sites; the amount of HIV DNA appeared to increase from duodenum to terminal ileum, right colon and rectum. In that study, sigmoid colon was not studied but the rectum was described as a major site for the persistence of cells harboring HIV DNA [35]. Yukl et al normalized viral loads to 10^6^ CD4+ T cells, and our data do not directly address the differences in assays. Although we cannot exclude that other sites of the GI tract might contribute to HIV persistence, the serial increase in HIV DNA from duodenum to rectum would suggest that sigmoid might be more akin to the latter, and we believe that the measurement of viral DNA in longitudinal sigmoid biopsy provides novel information by indicating that early ART greatly reduces the reservoir size in this compartment. Furthermore, we did not include information on integrated DNA as the values were extremely low and difficult to interpret for the small number of patients. The assay for 2LTR (long terminal repeat) circles is currently being optimized for CRF01_AE. Transient increases in 2LTR circles could occur as a result of raltegravir intensification [36]. Therefore, the total HIV DNA reported in our study may reflect non-integrated HIV DNA. In addition, we did not measure cell-associated HIV RNA (unspliced and multiply-spliced). Decreases in unspliced HIV RNA of ileal CD4+ T cells were reported in some patients who received raltegravir intensification [37]. Taken with these observations, the reduction of total DNA in sigmoid MMC at 24 weeks could be a lower bound estimate for integrated DNA.

Studying the immunologic and virologic changes during AHI could provide important insight that will inform the design of immunogens for preventive vaccines [5]. There may be a window of opportunity during early AHI to intervene and limit CD4 destruction and HIV reservoir formation with the ultimate goal of achieving drug-free remission of HIV. In addition to ART during early Fiebig stages, strategies such as therapeutic HIV vaccines or drugs that target the long-lived cellular reservoir may be necessary to achieve this goal [2], [38] – and persons aggressively treated in these early Fiebig stage infections may be ideal candidates for these interventions. In order to test for HIV functional cure, treatment interruption will be necessary. The risk for serious non-AIDS events due to treatment interruption seen in the SMART study is a concern [39]; however, this may be mitigated in early-treated AHI subjects who have preserved immunity and limited HIV reservoir [22], provided it is done in the setting of a closely monitored clinical trial.
